# Metanephric adenoma diagnosed on biopsy in an infant: a case report

**DOI:** 10.1186/s13256-023-04046-1

**Published:** 2023-08-08

**Authors:** S. Mosbahi, S. Ben Youssef, A. Zouaoui, M. Abdelali, M. Ben Fredj, N. Ben Abdejelil, S. Belhassen, S. Hidouri, I. Chabchoub, A. Ksia, L. Sahnoun, M. Mekki, A. Zakhama, A. Zrig, M. Belghith

**Affiliations:** 1 Department of Pediatric Surgery, Fattouma Bourguiba University Hospital Monastir, Faculty of Medicine of Monastir, University of Monastir, Farhat Hached Street, 5000 Monastir, Tunisia; 2 Department of Radiology, Fattouma Bourguiba University Hospital Monastir, Faculty of Medicine of Monastir, University of Monastir, Monastir, Tunisia; 3 Department of AnatomopathologyFattouma Bourguiba University Hospital Monastir, Faculty of Medicine of Monastir, University of Monastir, Monastir, Tunisia; 4 Department of Oncology, Farhat Hached University Hospital, Faculty of Medicine of Sousse, University of Sousse, Sousse, Tunisia

**Keywords:** Metanephric adenoma, Conservative surgery, Infant

## Abstract

**Background:**

Metanephric adenoma is a rare benign renal tumor of the kidney, uncommonly observed in children. It is often misdiagnosed preoperatively as a malignant neoplasm, leading to an unnecessary nephrectomy. The challenge is to make the right diagnosis preoperatively and therefore manage it with conservative surgery. We report a case of a child with metanephric adenoma who underwent nephron-sparing surgery.

**Case presentation:**

A renal tumor was discovered fortuitously in an 18-month-old Caucasian girl with several congenital malformations. Investigations showed a 28 × 27 × 27 mm left renal mass centrally located, well defined, nonvascularized, with no calcifications and which compressed the adjacent renal tissue. Furthermore, there were no signs of metastasis. The decision of a multidisciplinary meeting was to perform a computed tomography (CT)-scan-guided biopsy. Histologic examination concluded it was a metanephric adenoma. We performed a left open partial nephrectomy via a flank retroperitoneal incision. The final histopathological examination confirmed the diagnosis. The postoperative course was uneventful.

**Conclusion:**

Preoperative diagnosis of metanephric adenoma is challenging. Because of the high probability of unnecessary radical nephrectomy, preoperative biopsy can be safe and determining to guide a more conservative approach so nephron-sparing surgery can be performed.

## Background

Metanephric adenomas (MAs) are rare benign neoplasms of the kidney. They derive from the renal residual tissue during embryonic development. These tumors occur predominantly in middle-aged women with few cases reported in children [1, 2].

Because of the lack of specific clinical and radiographic characteristics, they are frequently misdiagnosed preoperatively as malignant tumors of the kidney. The diagnosis is mostly established based on histological examination after an unnecessary radical nephrectomy. However, accurate diagnosis made preoperatively is of great importance as MA should be treated conservatively with nephron-sparing surgery. Herein, we describe the case of an infant who underwent conservative treatment for a metanephric adenoma diagnosed by preoperative fine-needle biopsy.

## Case presentation

### Observation

An 18-month-old Caucasian girl was referred to our department after the fortuitous discovery of a renal mass. She presented with history of microcrania, cataract, and strabismus with no specific malformative syndrome. There was no family history of consanguinity, malformative or oncologic disease. She was operated on for her congenital cataract with uneventful course. She was treated by valproic acid for one episode of idiopathic epileptic crisis. On initial physical examination, hemodynamic constants and temperature were normal, we found a microcranium with cranial perimeter of 41 cm under 3 Standard Deviation (SD)  with nothing to notice on the rest of the neurological examination. There was also no abdominal tenderness nor a palpable mass. On biological assessment: hematology, electrolytes, renal, and hepatic functions were normal aside from a polycythemia. The activated partial thromboplastin time (APTT) or activated clotting time (ACT) was extended with a value of 63 s, prothrombin time was at 100%, and Rosner index was at 16.43%, which could be due to a lupus anticoagulant, although specific investigations ruled out this probability. Urine cytology and culture results were within normal limits, without hematuria. As part of an assessment for other malformations, an abdominal ultrasound was performed, which showed a mass arising within the left kidney.

### Investigation

Abdominal ultrasonography revealed a small left round solid central renal mass, hyperechoic, well defined, weakly vascularized without calcifications. It measured 26 × 28 mm.

**Fig. 1:**
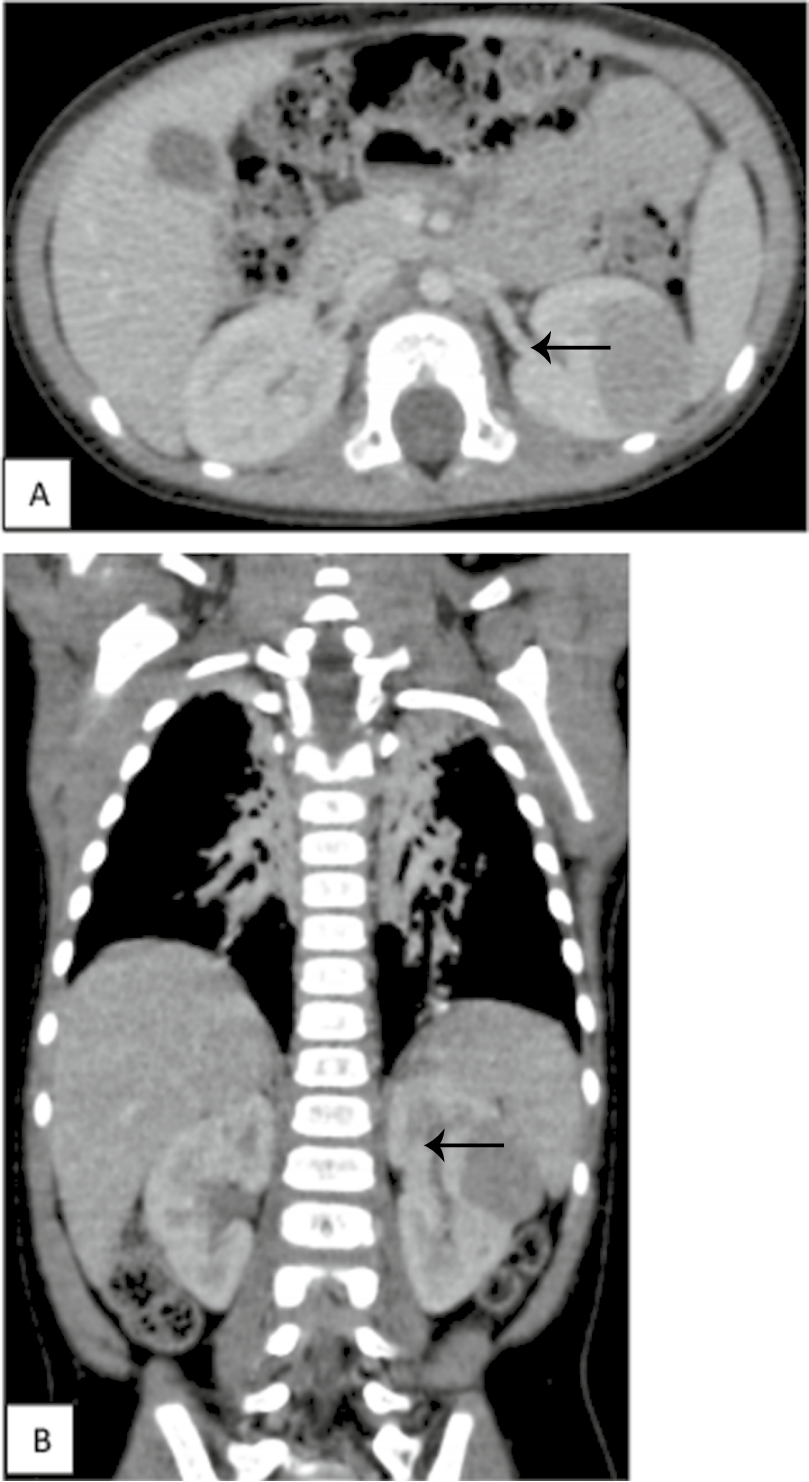
Abdominal CT scan in axial section (**A**) and coronal reconstruction (**B**) at portal time showing a tissue mass in the middle part of the left kidney with regular contours and which is enhanced homogeneously (← )

CT scan of the abdomen showed a small round subcapsular tumor, arising from the medial part of the left kidney (28 × 27 × 27 mm). It was well defined and compressed the adjacent renal tissue. The tumor was not clearly enhanced in the early phase on CT. There were some areas of necrosis, but there was no evidence of metastatic disease (Fig. 1). Wilms’ tumor was evoked, but the radiological characteristics of the mass were not conclusive enough. After an interdisciplinary discussion, the decision was to perform a CT-scan-guided biopsy of the tumor so that adequate treatment could be performed. The pathological examination concluded on a metanephric adenoma by showing a specimen composed of tightly packed tubules and ductular structures with little intervening stroma. There was no cytologic atypia. Mitotic figures were rare. There were no papillary areas. Knowing that nephron-sparing surgery could be safely performed thanks to the preoperative tumor biopsy findings, we decided to proceed with open left flank retroperitoneal conservative management.

### Treatment

A left flank retroperitoneal incision exposed the left kidney. The inspection showed a well-circumscribed nonencapsulated polylobed tumor. Its appearance was similar to that of normal kidney tissue. The tumor measured 30 × 20 × 30 mm with a noticeable cleavage plane. An easy wedge resection of the tumor was performed using the electric scalpel, ensuring perfect hemostasis and respecting the caliceal cavities.

**Fig. 2:**
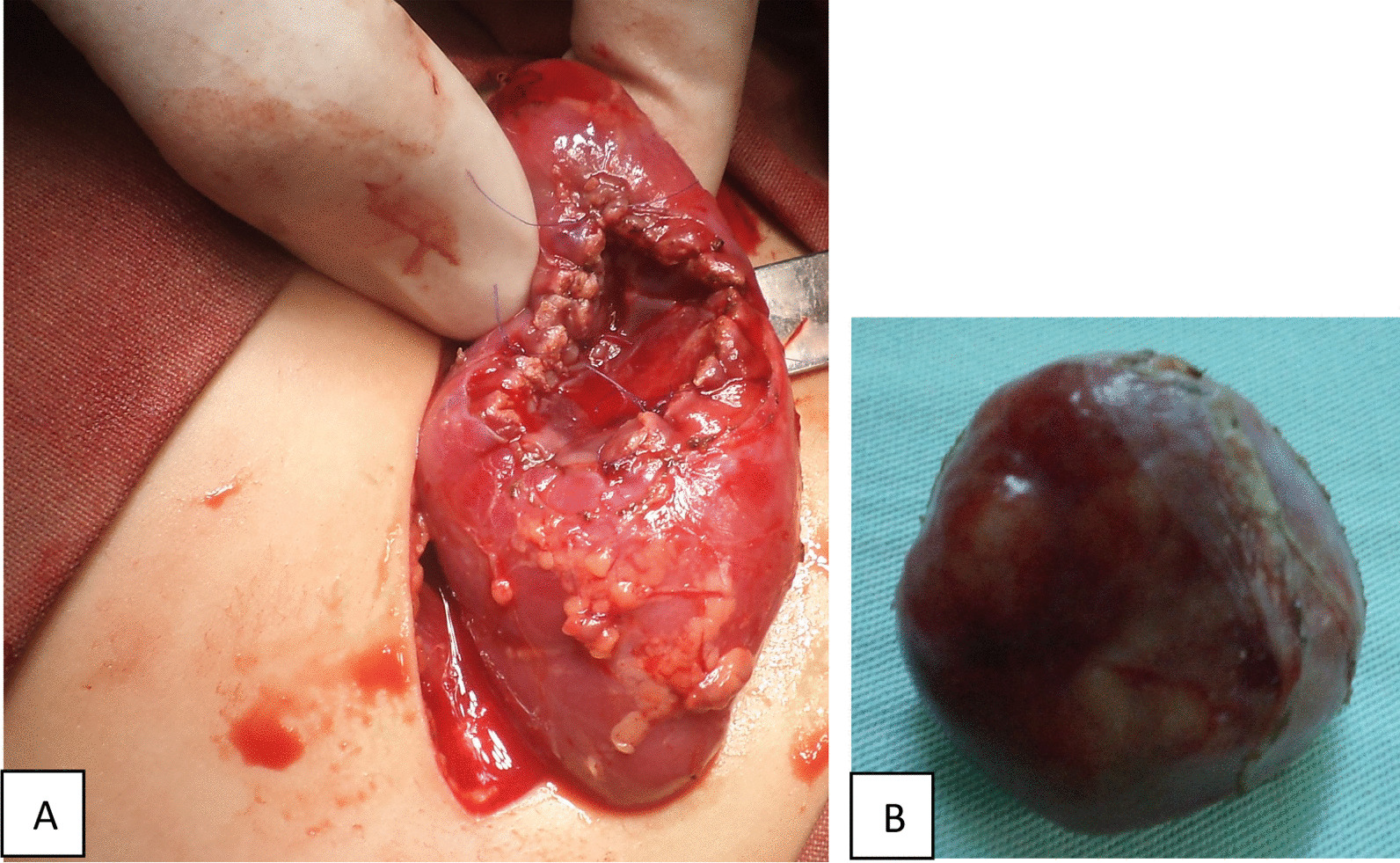
**A**, **B** Total resection of the kidney tumor

**Fig. 3:**
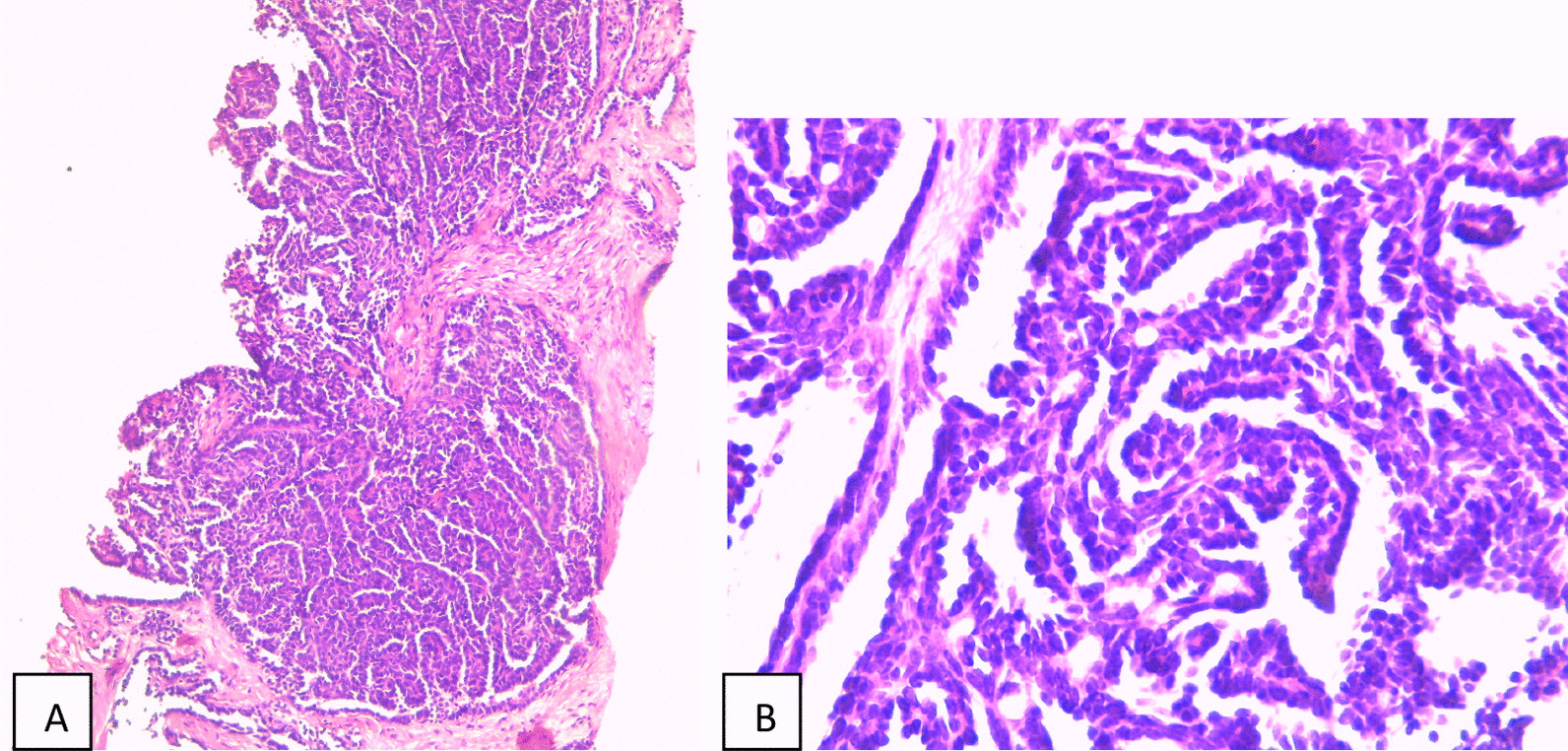
**A** Small, uniform, closely packed tubules and papillae in loose stroma (HE × 100). **B** The lining epithelial cells are uniform and small with hyperchromatic nuclei and scant cytoplasm (HE × 400)

Macroscopically, the tumor was well circumscribed and yellow–white and had an intact tegument with homogeneous and gray cutting surface (Fig. 2). Microscopically, on hematoxylin–eosin staining, the tumor was composed of small, uniform, tubules and acini in loose scant stroma. The lining epithelial cells were uniform and small with hyperchromatic rare and small nuclei and scant acidophilic cytoplasm. No mitotic activity or necrosis was present (Fig. 3). Wide clear surgical margins were identified between the kidney and the tumor. Immunohistochemical staining revealed positive immunoreactivity for vimentin and Wilms’ tumor 1 (WT1) and partially positive for CD56, CD57 and negative immunoreactivity for CK7, CK20, chromogranin, and synaptophysin. The final pathological diagnosis was metanephric adenoma of the left kidney.

### Outcomes and follow-up

Oncologists and surgeons recommended no further treatment. The patient had an uneventful postoperative course and was discharged on the fourth day. The patient was followed postoperatively by physical examination, abdominal ultrasound, and biological renal function every 3 months during the first year, and then every 6 months. The clinical, biological, and radiological follow-up was uneventful 4 years later.

## Discussion

Management of renal masses in children can be challenging, especially when lacking specific radiologic features. MA is a less common benign condition in children; its similarity to other malignant renal tumors makes establishing a preoperative diagnosis very important to guide the adequate surgery technique. To our knowledge, our patient is the youngest case of a child with MA who underwent nephron-sparing surgery [3].

Clinically, MA may present with hematuria, flank pain, hypertension, or abdominal mass [3–5]. However, as in this case, metanephric adenomas are usually asymptomatic lesions, detected incidentally on imaging studies performed for other indications. However, imaging can only offer some general clues that can be used only to suspect the diagnosis of MA. CT is the main imaging method used for diagnosis, but there is no specific radiological feature of MA. Generally, MA is consistently well defined and mostly has an intact capsule with no distinct attenuation patterns. It is mostly spontaneous and slightly hyperdense in comparison with normal renal parenchyma [6]. Calcifications can be observed in 20% of cases [7]. Delzongle *et al.* [5] concluded that there was no correlation between CT or magnetic resonance imaging (MRI) findings and histological characteristics of MA and that imaging cannot be specific at all. Those findings led to the approach of preoperative biopsy to improve diagnostic accuracy and spare the children an unnecessary nephrectomy. Amodio *et al.* [3] reported that MA can be confused with other neoplasms such as sarcoma or carcinoma and is hardly detectable without preoperative biopsy. Fine-needle biopsy in the management of small renal masses is still rather controversial. In fact, surgical management based on CT imaging without pretreatment biopsy is considered appropriate in most centers. However, 20% of surgically removed small renal tumors have proven to be benign [8]. Thus, it is necessary for current practices to be reevaluated to avoid overtreatment. Actually, fine-needle percutaneous biopsy is considered a safe and effective procedure in the management of small renal masses [9].

The final diagnosis is confirmed by postoperative histopathological examination, which accurately describes all the characteristics of the tumor [10]. Microscopically, the classic appearance of MA is a cellular blue tumor composed of tightly packed tubules, long branching and angulated ducts, and abortive glomeruli. Tumor cells have a scant cytoplasm and small nuclei with no nucleoli. Mitotic figures are very rare or absent. Stroma is scant and can be edematous and occasionally look scar-like. Psammomatous calcifications can be abundant [11]. Differential diagnoses include papillary renal cell carcinoma type 1, solid variant and adult epithelial predominant nephroblastoma. Papillary renal cell carcinoma, type 1 is a PAX8+ , vimentin+ , CK7+ , AMACR+ , WT1−, CD57−, BRAF− tumor as opposed to metanephric adenoma that is positive for WT1, CD57, and BRAF but negative for CyK7. Adult nephroblastoma is positive for WT1 but negative for CD57 and BRAF [11]. In our case we did not test BRAF due to a lack of this antibody in our Anatomopathology department.

Partial or radical nephrectomy is the mainstay of treatment for MA. Radical nephrectomy still remains the treatment of choice when the preoperative biopsy is not conclusive.

The possibility of nephron-sparing surgery in MA in the pediatric population has previously been addressed in literature [12]. Partial nephrectomy or thermoablative procedures are also recommended, according to the American Urological Association Guidelines [13].

The follow-up is not well defined, but should be done with clinical and radiological examination according to Liniger *et al.* [10].

## Conclusion

MA is rare in children, and even rarer in infants. Its preoperative diagnosis is challenging as it lacks specific imaging features. Giving the high probability of subjecting infants to an unnecessary radical nephrectomy, preoperative biopsy can be a safe procedure that guides the management plan toward the more conservative nephron-sparing surgery.
